# Supplementary figures and images for: sigNATURE maps cohort-specific T-cell states to reproducible programs of ICI response
Source: bioRxiv. 2026 Apr 15:2026.04.14.718532. Preprint. [Version 1] doi: 10.64898/2026.04.14.718532 (PMC13104942; doi:10.64898/2026.04.14.718532)

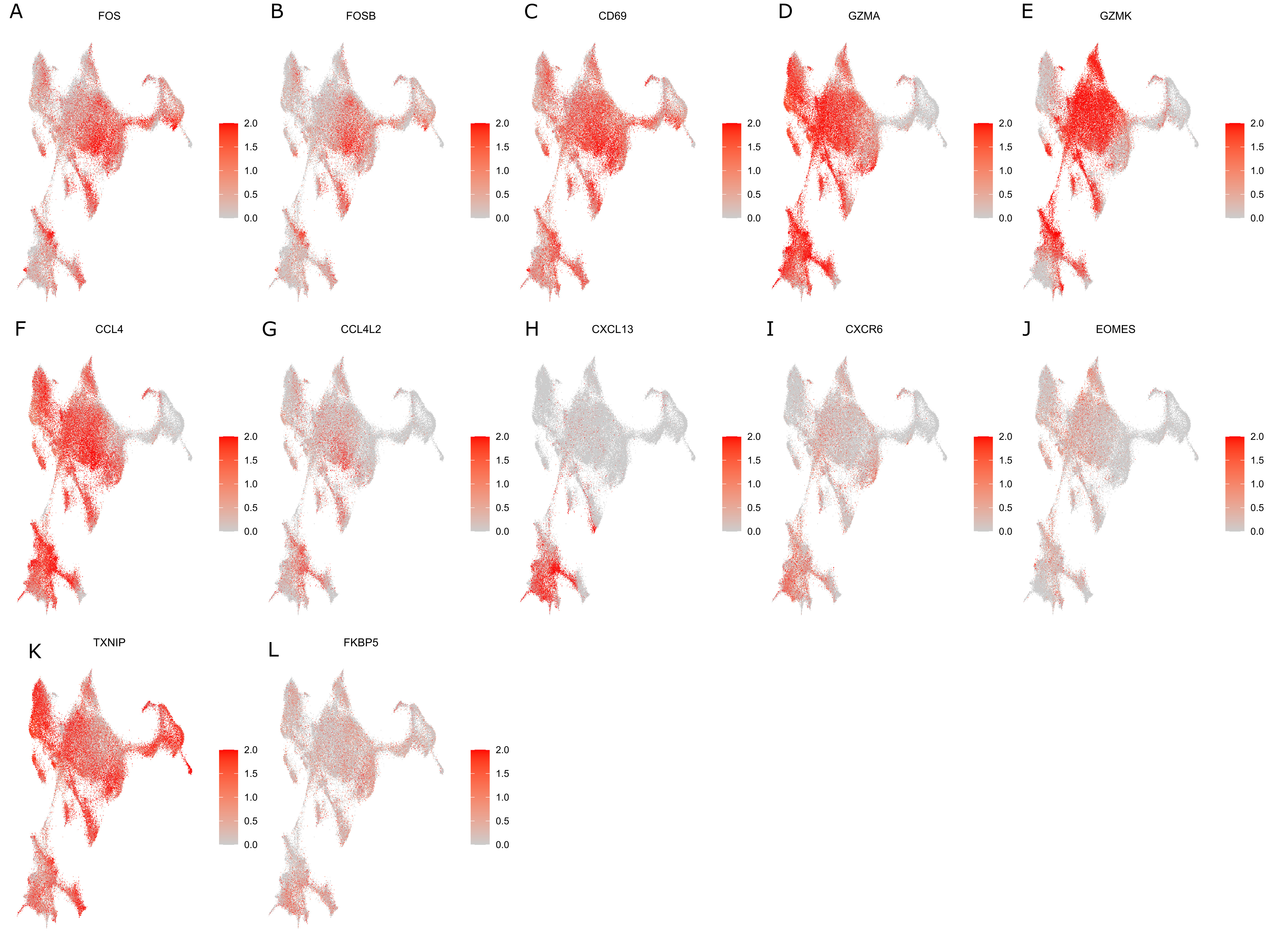

Supplement: Supplement 1 — S. Fig. 1 Expression UMAP of (A) FOS, (B) FOSB, (C) CD69, (D) GZMA, (E) GZMK, (F) CCL4, (G) CCL4L2, (H) CXCL13, (I) CXCR6, (J) EOMES, (K) TXNIP, (L) FKBP5 on sigNATURE CD8+ reference atlas. [file media-1.jpg]

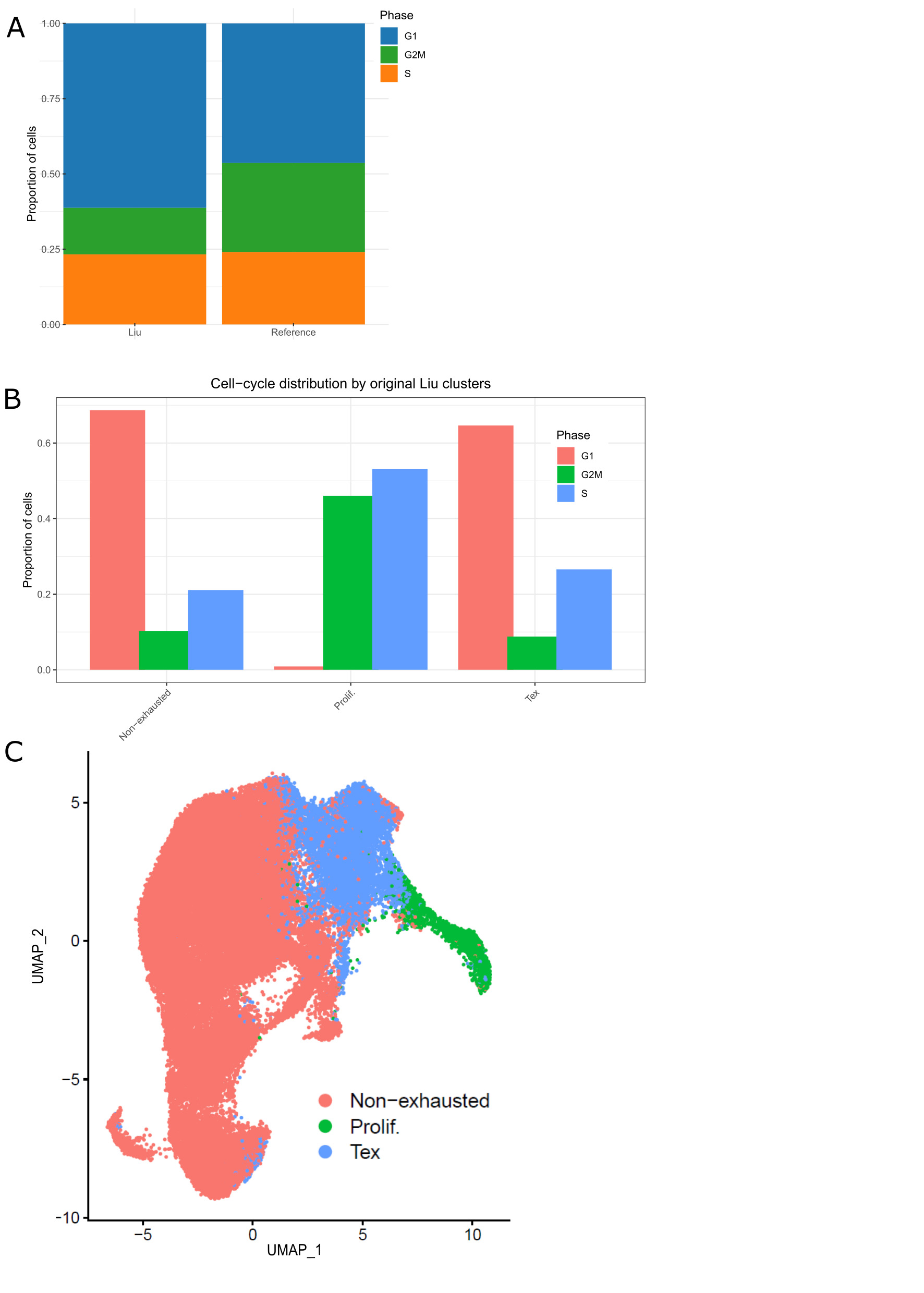

Supplement: Supplement 2 — S. Fig. 2 (A) Cell proportion of Liu et al’s and our CD8 reference data in terms of cell cycle information, whether it is G1, G2M, or S. (B) Cell proportion of Liu et al’s CD8 T cell states in terms of cell cycle information, whether it is G1, G2M, or S. (C) UMAP of Liu et al.’s T cell clusters before cell-cycle normalization [file media-2.jpg]

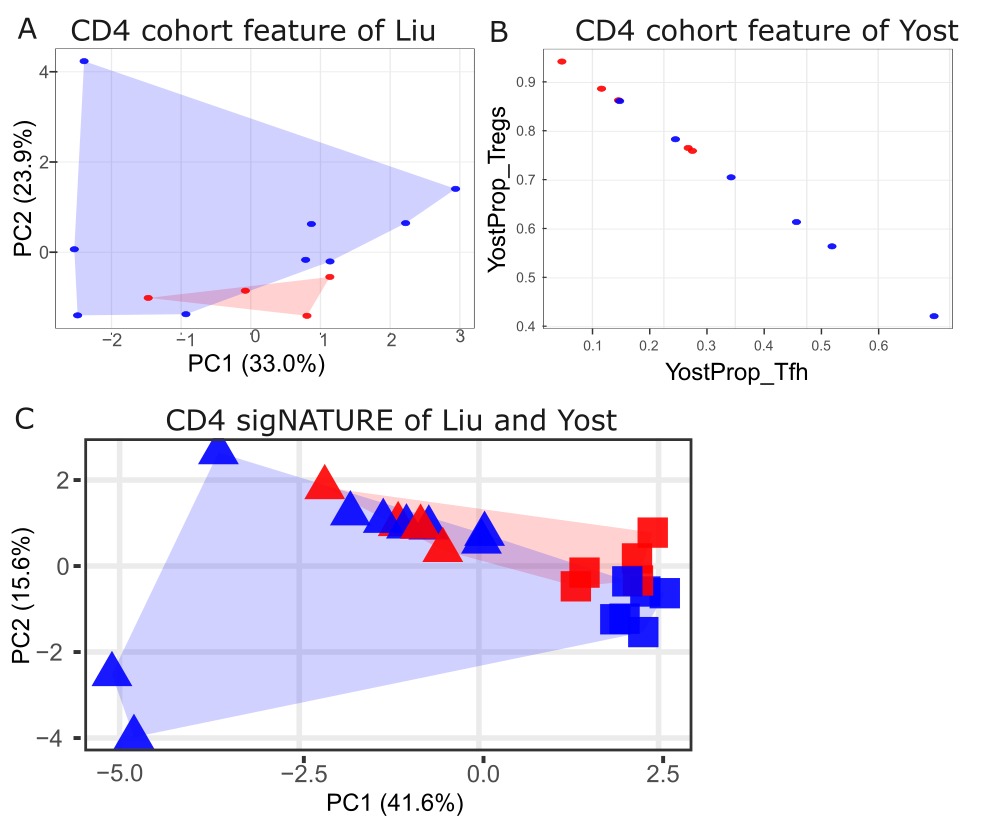

Supplement: Supplement 3 — S. Fig. 3 (A, B) Low-dimensional projections of cohort-derived CD4 or CD8 T-cell features from Liuthe Liu et al. and Yost et al. cohorts, showing substantial overlap between responders and non-responders. (C) Low-dimensional projection of sigNATURE-derived features showing improved separation of response groups and enabling direct comparison of independent cohorts in a shared atlas-aligned state space. [file media-3.jpg]
